# Improvement of electric field-induced strain and energy storage density properties in lead-free BNKT-based ceramics modified by BFT doping

**DOI:** 10.1039/c9ra00956f

**Published:** 2019-04-16

**Authors:** Pharatree Jaita, Ratabongkot Sanjoom, Narumon Lertcumfu, Gobwute Rujijanagul

**Affiliations:** Department of Physics and Materials Science, Faculty of Science, Chiang Mai University Chiang Mai 50200 Thailand rujijanagul@yahoo.com; Science and Technology Research Institute, Chiang Mai University Chiang Mai 50200 Thailand; Department of Applied Science and Biotechnology, Faculty of Agro-Industrial Technology, Rajamangala University of Technology Tawan-ok Chanthaburi Campus Chanthaburi 22210 Thailand

## Abstract

In this research, the effects of Ba(Fe_0.5_Ta_0.5_)O_3_ (BFT) additive on the phase evolution, the dielectric, ferroelectric, piezoelectric, electric field-induced strain responses, and energy storage density of the Bi_0.5_(Na_0.80_K_0.20_)_0.5_TiO_3_–0.03(Ba_0.70_Sr_0.03_)TiO_3_ (BNKT–0.03BSrT) ceramics have been systematically investigated. The ceramics have been prepared by a solid-state reaction method accompanied by two calcination steps. X-ray diffraction indicates that all ceramics coexist between rhombohedral and tetragonal phases, where the tetragonal phase becomes dominant at higher BFT contents. The addition of BFT also promotes the diffuse phase transition in this system. A significant enhancement of electric field-induced strain response (*S*_max_ = 0.42% and 
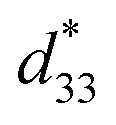
 = 840 pm V^−1^) is noted for the *x* = 0.01 ceramic. Furthermore, the giant electrostrictive coefficient (*Q*_33_ = 0.0404 m^4^ C^−2^) with a giant normalized electrostrictive coefficient (*Q*_33_/*E* = 8.08 × 10^−9^ m^5^ C^−2^ V^−1^) are also observed for this composition (*x* = 0.01). In addition, the *x* = 0.03 ceramic shows good energy storage properties, *i.e.* it has a high energy storage density (*W* = 0.65 J cm^−3^ @ 120 °C) with very high normalized storage energy density (*W*/*E* = 0.13 μC mm^−2^), and good energy storage efficiency (*η* = 90.4% @ 120 °C). Overall, these results indicate that these ceramics are one of the promising candidate piezoelectric materials for further development for actuator and high electric power pulse energy storage applications.

## Abstract 2

The effects of Ba(Fe_0.5_Ta_0.5_)O_3_ additive on phase, dielectric, ferroelectric, piezoelectric, electric field-induced strain, and energy storage density of the Bi_0.5_(Na_0.80_K_0.20_)_0.5_TiO_3_–0.03(Ba_0.70_Sr_0.03_)TiO_3_ ceramics have been investigated.

## Graphical abstract

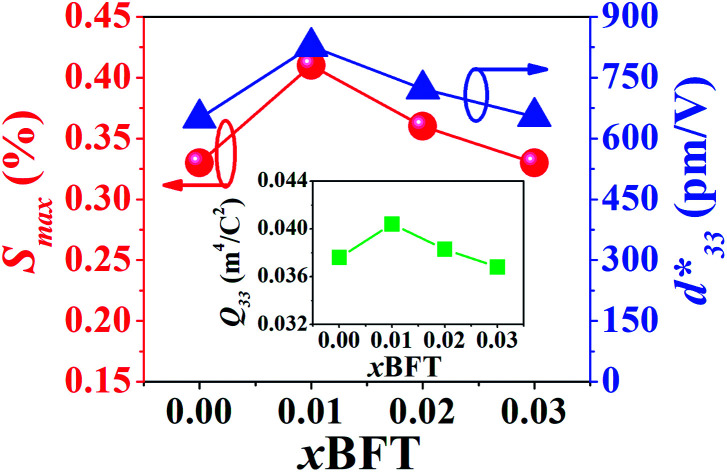

## Introduction

For actuator applications, electrostriction is an important property which describes how the material deforms when polarization develops inside.^1^ Electrostriction occurs in materials with either centro or noncentrosymmetric crystal structures, and is caused by a slight displacement of ions (or complexes) in the crystal lattice when the material is subjected to an applied field.^2^ In the case of electrostriction, the field-induced strain (*S*) can be simply related to the polarization (*P*) by: *S* = *Q*_33_*P*^2^, where *Q*_33_ represents the electrostrctive coefficient.^2–5^ A large *Q*_33_ value of ∼0.020 m^4^ C^−2^ with a good hysteresis-free electrostrictive strain (∼0.1%) can be observed in some lead-based relaxor ferroelectrics, such as Pb(Mg_1/3_Nb_2/3_)O_3_ (PMN),^3^ which is known as an important electrostrictive material.^5^ However, because of the concern for environmental problems that lead-based materials pose, the development of lead-free electrostrictive piezoelectric materials has been urgent in recent years.^3,5,6^

Among many lead-free piezoelectric materials, (Bi_0.5_Na_0.5_)TiO_3_-based (BNT) ceramic is an interesting material due to its relatively excellent electrical properties, especially for ferroelectric and piezoelectric properties^7^*i.e.* high electric energy densities^8^ and ultra-high strain under a high electric field (≥50 kV cm^−1^).^9^ However, the large coercive electric field (*E*_c_ = 7.3 kV mm^−1^) and high electrical conductivity made it difficult to be polarized during the poling process, which often leads to undesirable piezoelectricity (*d*_33_ = 73–95 pC N^−1^) and limited its applications.^10^ In order to reduce the *E*_c_ and/or improve the electrical properties, researches on composition-modified BNT systems have been conducted such as BNT–BT,^11,12^ BNT–ST,^13^ BNT–BZT–SBT,^14^ and BNT–BKT.^15,16^ Ullah *et al.*^17^ studied the effect of (Ba_0.70_Sr_0.30_)TiO_3_ (BST) on the structure and electrical properties of the Bi_0.5_(Na_0.40_K_0.10_)TiO_3_ (BNKT) ceramic. They reported that this ceramic exhibited good electrical properties *i.e.* the ceramics presented high piezoelectric coefficient (*d*_33_ = 223 pC N^−1^) with dielectric loss (tan *δ*) of 3% at 1 kHz. They also found that the La additive interrupted the *P*–*E* hysteresis loops of the BNKT–BST ceramics, which lead to a reduction in *P*_r_ and *E*_c_ values.^18^ However, the destabilization of the ferroelectric order corresponded to a significant increase of the unipolar strain which showed the highest value of ∼0.39% and corresponding a normalized strain 
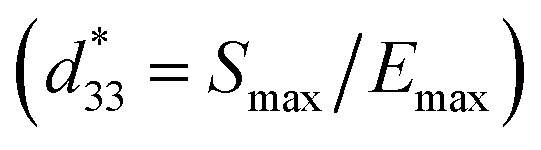
 of 650 pm V^−1^.^18^ Therefore, research on modified BNKT–BST ceramics is an important topic in order to develop lead-free ceramics with high electrical properties.

An interesting work for electrostrictive materials was performed by Zhang *et al.*^19^ who provided a research direction to develop the (1 − *x*)[0.94Bi_0.5_Na_0.5_TiO_3_–0.06BaTiO_3_]–*x*K_0.5_Na_0.5_NbO_3_ system with a large electrostrictive coefficient (*Q*_33_ ∼ 0.021–0.027 m^4^ C^−2^) and the maximum strain (*S*_max_ ∼ 0.12–0.30%) values simultaneously. Beside Zhang *et al.*'s work, a large electrostrictive effect with *Q*_11_ = 0.0297 m^4^ C^−2^ was also obtained by Shi *et al.*^20^ in the (0.94 − *x*)(Bi_0.5_Na_0.5_)TiO_3_–0.06BaTiO_3_–*x*(Sr_0.7_Bi_0.2_□_0.1_)TiO_3_ system, in which its *Q*_11_ exhibits a high temperature stability between ambient temperature to 180 °C.^20^ Moreover, a high strain (*S*_max_ ∼ 0.45%) was also found in the (Bi_0.5_Na_0.5_)TiO_3_–BaTiO_3_–(K_0.5_Na_0.5_)NbO_3_ or BNT–BT–KNN ceramic by Zhang *et al.*,^21^ which is even higher than the strain value obtained in the lead ferroelectric Pb(Zr,Ti)O_3_ ceramic. However, due to the phase evolution in BNT-based materials, large strain hysteresis larger than 60% can be observed inherently, which restricts the practicability use as electrostrictive materials.^4^ Thus research on the reduction of the strain hysteresis for the lead-free materials such as BNKT–BST based materials is an interesting issue for the lead-free actuators.

Recently, many authors have suggested that the iron oxide such as Fe_2_O_3_ additive can improve the piezoelectric and magnetic properties of some lead-free piezoelectric ceramics.^22,23^ In the case of piezoelectric properties, Jaita *et al.*^23^ reported that the Fe_2_O_3_ additive can enhance the electric field-induced strain of BNKT-based ceramics. However, the electrostriction and electric field-induced strain properties of lead-free piezoelectric materials such as BNT-based ceramics doped with complex perovskite materials containing Fe have not been widely investigated. For the Fe-containing complex perovskite material, *i.e.* barium iron tantalate (BaFe_0.5_Ta_0.5_O_3_; BFT) is interesting because it exhibits a giant dielectric material which has attracted great attention due to its very high dielectric constant (*ε*_r_ ∼ 1.9 × 10^5^ at 550 °C and 1 kHz).^24^ Many works have reported that BFT, which was first synthesized by Galasso *et al.*,^25^ presents a cubic perovskite-type structure with space group *Pm*3̄*m* (221)^24–26^ and the lattice parameter *a* = 4.056 Å.^24,27^ BFT can be used to modify other materials such as BiFeO_3_ ^24^ and Ba(Zr_0.05_Ti_0.95_)O_3_ (BZT),^27^ and the properties of the modified ceramics have been improved. For example, the dielectric constants of the BZT–BFT solid solution were enhanced by BFT.^28^

In the present research, new lead-free of Bi_0.5_(Na_0.80_K_0.20_)_0.5_TiO_3_–0.03(Ba_0.70_Sr_0.03_)TiO_3_ doped with Ba(Fe_0.5_Ta_0.5_)O_3_ ceramics were synthesized with the aim of improving their electrical properties. The role of BFT content on the ceramic properties including phase formation, microstructure, electrical properties (*i.e.* dielectric, ferroelectric and piezoelectric, and the electric field-induced strain behavior) of the BNKT–0.03BSrT ceramic were investigated. Furthermore, since many recent works have focused on energy storage materials due to global energy problems, thus the storage energy density behavior of the presented ceramics was also investigated.

## Experimental

The conventional mixed oxide technique was used to synthesize the (1 − *x*)[Bi_0.5_(Na_0.80_K_0.20_)_0.5_TiO_3_–0.03(Ba_0.70_Sr_0.03_)TiO_3_]–*x*Ba(Fe_0.5_Ta_0.5_)O_3_ or (1 − *x*)[BNKT–0.03BSrT]–*x*BFT powders. The analytical grade reagents of metal oxide powders, including Na_2_CO_3_, Bi_2_O_3_, K_2_CO_3_, TiO_2_, Fe_2_O_3_, SrCO_3_, BaCO_3_, and Ta_2_O_5_ were used as starting materials. All carbonate powders were first dried at 120 °C for 24 h in order to eliminate any moisture. The raw materials of BNKT–0.03BSrT and BFT were stoichiometrically weighed, ball milled for 24 h in an ethanol solution, and then dried in an oven. Since there is a large difference in calcination temperatures between BNKT–0.03BSrT (900 °C) and BFT (1200 °C) powders, two calcination steps were employed *i.e.* the BNKT–0.03BSrT and BFT powders were synthesized separately. The resulting powders were weighed, mixed and then dried to obtained a powder of (1 − *x*)[BNKT–0.03BSrT]–*x*BFT with *x* = 0, 0.0, 0.02 and 0.03 mol fraction. A few drops of polyvinyl alcohol (PVA) binders (4 wt%) were added to the obtained powders before being uniaxially pressed into discs 10 mm in diameter. The green pellets were sintered at 1125 °C for 2 h by using a heating and cooling rate of 5 °C min^−1^.

Bulk density was determined by the Archimedes' method. A scanning electron microscope (SEM, JEOL JSM-6335F) was used to study microstructural features of the ceramics. An X-ray diffractometer (XRD-Phillip, X-pert) was used to study the phase evolution of all ceramics. Grain size of the ceramics was carried out by using linear intercept method (ASTM no. E112-88). Before investigating the electrical measurements, all samples were polished into a parallel surface with 1 mm thickness. Silver paste was subjected onto both sides of the sample. Then the samples were heated at 700 °C for 30 min to form electrodes. Dielectric properties as a function of temperature (25–400 °C) were carried out using a LCR-meter (HP model 4192A) at frequencies ranging from 1 to 1000 kHz. The ferroelectric properties were carried out by a Radiant Precision ferroelectric tester both at room temperature (RT) and high temperatures (HT) of 25–150 °C. A maximum electric field of 50 kV cm^−1^ at a frequency of 1 Hz was applied to each studied sample. Remanent polarization (*P*_r_), maximum polarization (*P*_max_), and coercive field (*E*_c_) values were determined from the hysteresis loops or *P*–*E* loops. By using data from ferroelectric properties, the energy storage density (*W*) and energy storage coefficient (*η*) values were also calculated. Strain–electric field (*S*–*E*) data at RT were carried out using an optical displacement sensor (Fotonic Sensor model MTI-2100) injunction with a Radiant ferroelectric test system. A maximum electric field of 50 kV cm^−1^ and a frequency of 0.1 Hz were used to measure the bipolar and unipolar strain curves. The maximum strain (*S*_max_) and the negative strain (*S*_neg_) values were carried out from the bipolar curve. The normalized strain coefficient 
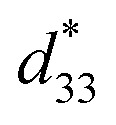
 was also determined following: 
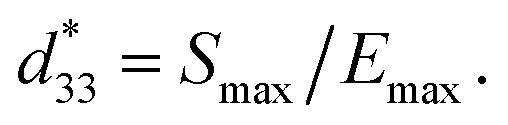
 The electrostrictive coefficient (*Q*_33_) was determined from *S*–*P* curves using equation: *S* = *QP*^2^. For piezoelectric measurement, all samples were poled at RT with an applied DC electric field of 5 kV mm^−1^ (for 15 min) and using silicone oil as the media. The low-field piezoelectric coefficient (*d*_33_) was determined by a *d*_33_-meter.

## Results and discussion

### Phase formation and microstructure

**Fig. 1:**
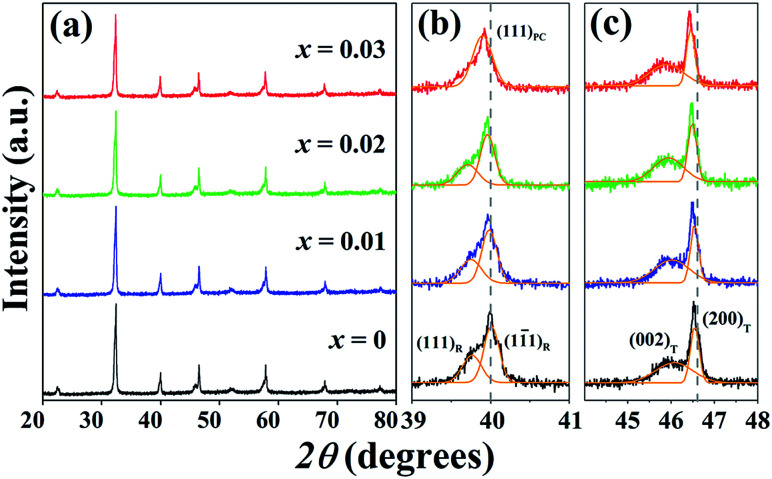
X-ray diffraction patterns of the (1 − *x*)[BNKT–0.03BSrT]–*x*BFT ceramics where (a) 2*θ* = 20–80° (b) 2*θ* = 39–41° and (c) 2*θ* = 44–48°.

**Fig. 2:**
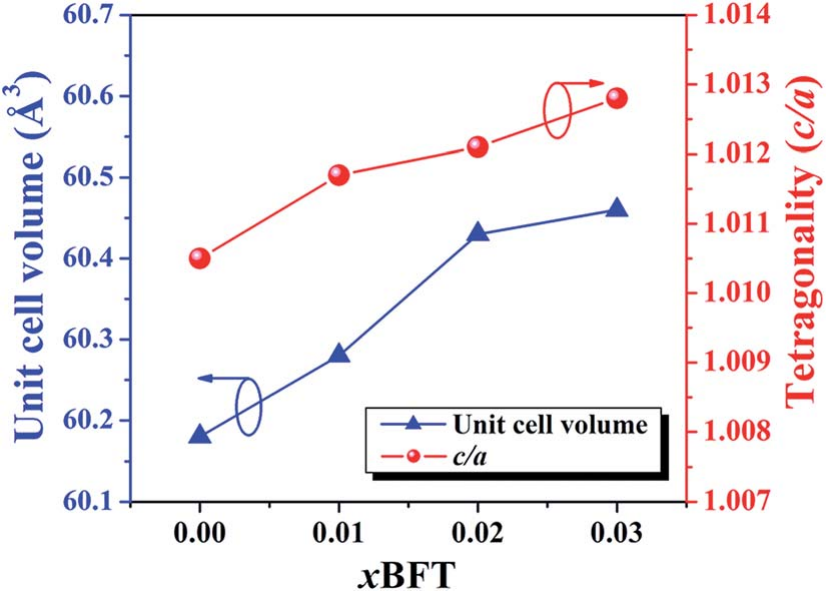
Plots of unit cell volume and the tetragonality (*c*/*a*) as functions of BFT content of the (1 − *x*)[BNKT–0.03BSrT]–*x*BFT ceramics.

Fig. 1(a) shows the X-ray diffraction patterns with different doping contents of the (1 − *x*)[BNKT–0.03BSrT]–*x*BFT ceramics. Within the resolution limit of XRD, all ceramics exhibit a single phase of perovskite structure with no secondary phase, confirming that BFT has been incorporated into the BNKT–0.03BSrT lattice to form solid solutions of the end compounds. For analyzing the phase transition process, the XRD patterns for selected narrow angular ranges of *2θ* = 39–41° and *2θ* = 44–48° are presented in Fig. 1(b) and (c), respectively. The *x* = 0 ceramic presents a mixed phase of rhombohedral and tetragonal structure, which is demonstrated by a slight splitting of the (111)_R_/(11̄1)_R_ at *2θ* ∼ 40° and splitting of the (200)_T_/(002)_T_ peaks at *2θ* ∼ 46°. This result also agrees with the result observed by Ge *et al.*^6^ However, the coexistence of the mixed rhombohedral and tetragonal phases transforms into a tetragonal-rich phase at higher BFT contents. This can be evidenced by, the existence of the splitting (200)_T_/(002)_T_ peaks, and the merger of the (111)_R_/(11̄1)_R_ peak into a single (111)_PC_ peak, thus indicating that the amount of tetragonal phase is higher than that of the rhombohedral phase, with increasing BFT content. To check the appearance of the phase transformation in more detail, the tetragonality ratio (*c*/*a*) was determined and its values are listed in Table 1. The *c*/*a* value increases from 1.0105 for the *x* = 0 ceramic to 1.0128 for the *x* = 0.03 ceramic, as expected. Furthermore, the diffraction peaks gradually shift to a lower angle with increasing BFT content. This can be caused by the differences in the ionic radii between Na^+^, Bi^3+^, K^+^, Sr^2+^, and Ba^2+^ at the A-site and Ti^4+^, Fe^3+^, and Ta^5+^ at the B-site^29^ which, as a result, can induce a structural distortion such as an enlargement of unit cell size, as shown in Fig. 2. A similar peak slightly shifted by partial substitution of Ba^2+^ for [Bi_0.5_(Na_0.80_K_0.20_)]^2+^ and Zr^4+^ for Ti^4+^ was reported by Chen *et al.*^30^ in the BNKT–BZT system. They also found that the (110) peak shifted to lower *2θ* angles could produce an increase in the lattice constant and the unit cell dimension with increasing BZT fraction.

**Table 1:** Phase, physical, microstructure and dielectric properties of the (1 − *x*)[BNKT–0.03BSrT]–*x*BFT ceramics

*x*	Density (g cm^−3^)	Relative density (%)	*c*/*a*	Unit cell volume (Å^3^)	Grain size (μm)	*T*_m_ (°C)	*ε*_max_ (@1 kHz)	*δ*_γ_ a (K)	*δ*_γ_ b (K)
0	5.80	98	1.0105	60.18	0.61	316	5156	83	73
0.01	5.83	98	1.0117	60.28	0.59	303	5151	91	94
0.02	5.85	99	1.0121	60.43	0.58	292	4814	94	117
0.03	5.86	99	1.0128	60.46	0.56	284	4716	105	123

a Poled sample.

b Unpoled sample.

**Fig. 3:**
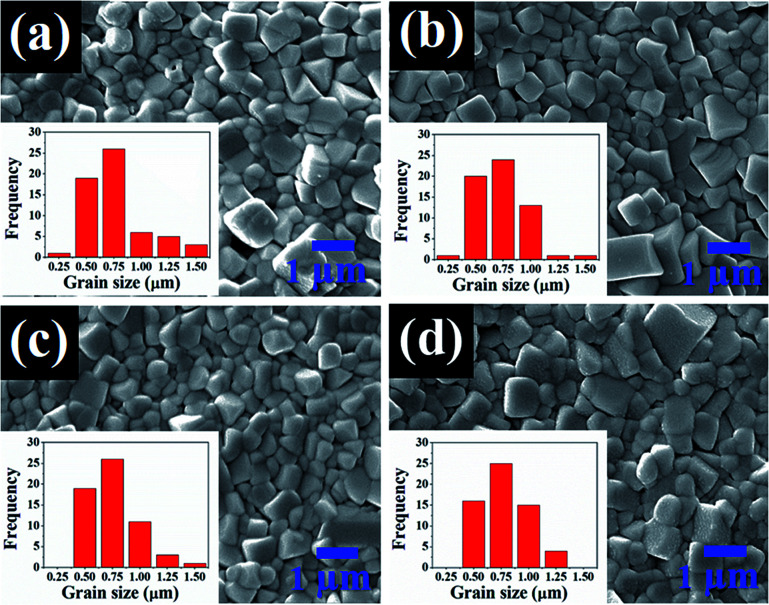
SEM micrographs and their corresponding histograms of grain size distribution of the (1 − *x*)[BNKT–0.03BSrT]–*x*BFT ceramics where (a) *x* = 0, (b) *x* = 0.01, (c) *x* = 0.02, and (d) *x* = 0.03.

SEM micrographs and their corresponding histogram of grain size distribution of the (1 − *x*)[BNKT–0.03BSrT]–*x*BFT ceramics are shown in Fig. 3. The average grain size values are also summarized in Table 1. The SEM result confirms that all samples are dense with relatively high densities (5.80–5.86 g cm^−3^). Furthermore, the porosity levels evident in the micrographs are noted to be consistent with the trend of measured density value, where the density increases with increasing BFT content. Most grains of all samples show a clear grain boundary with round and cubic shapes. The addition of BFT seems to have a slight influence on the microstructure as well as the average grain size of the BNKT–BSrT ceramics. The average grain size values of all samples are rather similar (0.56–0.61 μm) and the grain size distribution shows a monomodal normal distribution. However, a slight narrow grain size distribution is noted for a ceramic containing a higher amount of BFT. This suggests that the microstructure can be improved by a BFT additive.

### Dielectric properties

**Fig. 4:**
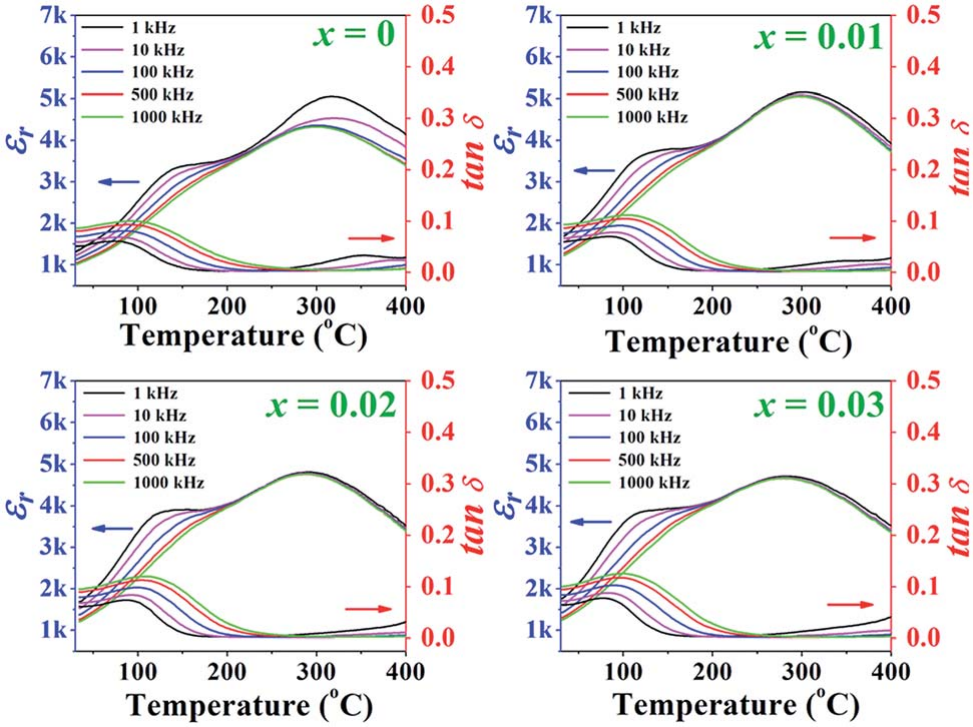
Temperature dependence of the dielectric constant (*ε*_r_) and dielectric loss (tan *δ*) of the poled (1 − *x*)[BNKT–0.03BSrT]–*x*BFT ceramics measured at various frequencies from 1–1000 kHz where *x* = 0–0.03.

Temperature dependence of the dielectric properties, including constant (*ε*_r_) and dielectric loss (tan *δ*) of the poled (1 − *x*)[BNKT–0.03BSrT]–*x*BFT ceramics measured at different frequencies from 1–1000 kHz are shown in Fig. 4, and the related values are also listed in Table 1. It can be seen that the *ε*_r_*versus* temperature (*ε*_r_–*T*) curves of all samples are rather similar. The *ε*_r_–*T* curves for all ceramics exhibit two dielectric anomaly peaks at ∼100 °C and 300 °C.^31–33^ Normally, the high temperature anomaly peak is called *T*_m_, where the dielectric constant reaches its maximum value.^34–36^ The lower anomaly peak is located near *T*_F–R_, which is known as the ferroelectric (FE) to ergodic relaxor (ER) phase transition temperature.^6^ Normally, the *T*_F–R_ can be usually carried out from the peak of tan *δ*–*T* curve of a poled sample. However, the *T*_F–R_ could not be detected from the tan *δ*–*T* curves for this work. The *T*_m_ and *ε*_max_ values of the *x* = 0 ceramic in this study are observed from Fig. 4 to be 316 and 5156, respectively. The *ε*_max_ decreases with increasing BFT content. Furthermore, the *T*_m_ also shifts from 316 °C for the *x* = 0 ceramic to 284 °C the *x* = 0.03 ceramic. This result also agrees with the results of previous report for other BNKT-based ceramics.^34^

To study the diffuse phase transition in this system, the diffuseness of the phase transition was calculated by a modified Curie–Weiss law eqn (1) which is given by ref. 37 and 38.1
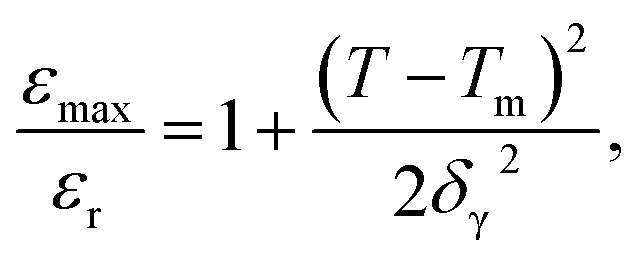
where *δ*_γ_ is the diffuseness parameter. The *δ*_γ_ value can be carried out from the curve of ln(*ε*_max_/*ε*_r_ − 1) *vs.* ln(*T* − *T*_m_)^2^, where the curve should be linear.^37^ The *δ*_γ_ is used to compare the degree of diffuseness of the phase transition for many materials. The *δ*_γ_ values for poled and unpoled ceramics are summarized in Table 1. Based on Table 1, it can be seen that the addition of BFT has an effect on the degree of diffuseness, where the *δ*_γ_ value increases with increasing BFT content. This result corresponds to the previous work,^39^ which indicates that the addition of BFT produces a higher degree of diffuseness for the phase transition in the modified BNKT ceramics.

### Polarization and strain behaviors

**Fig. 5:**
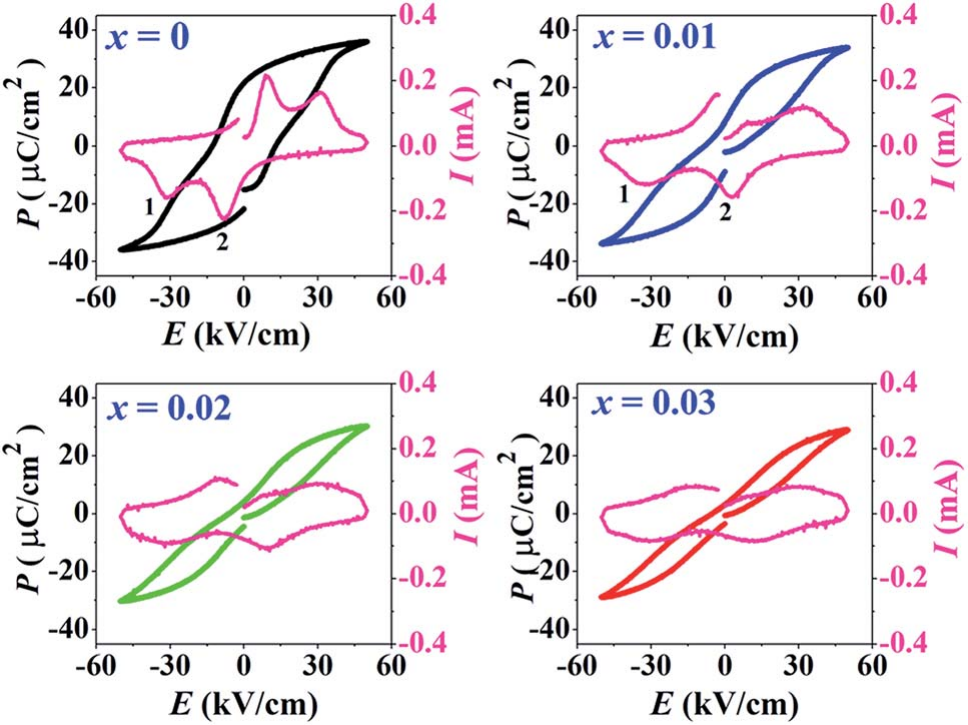
Polarization–electric field (*P*–*E*) hysteresis loops and current–electric field (*I*–*E*) loops of the (1 − *x*)[BNKT–0.03BSrT]–*x*BFT ceramics where *x* = 0–0.03, measured at RT under an electric field of 50 kV cm^−1^ and a frequency of 1 Hz.

**Fig. 6:**
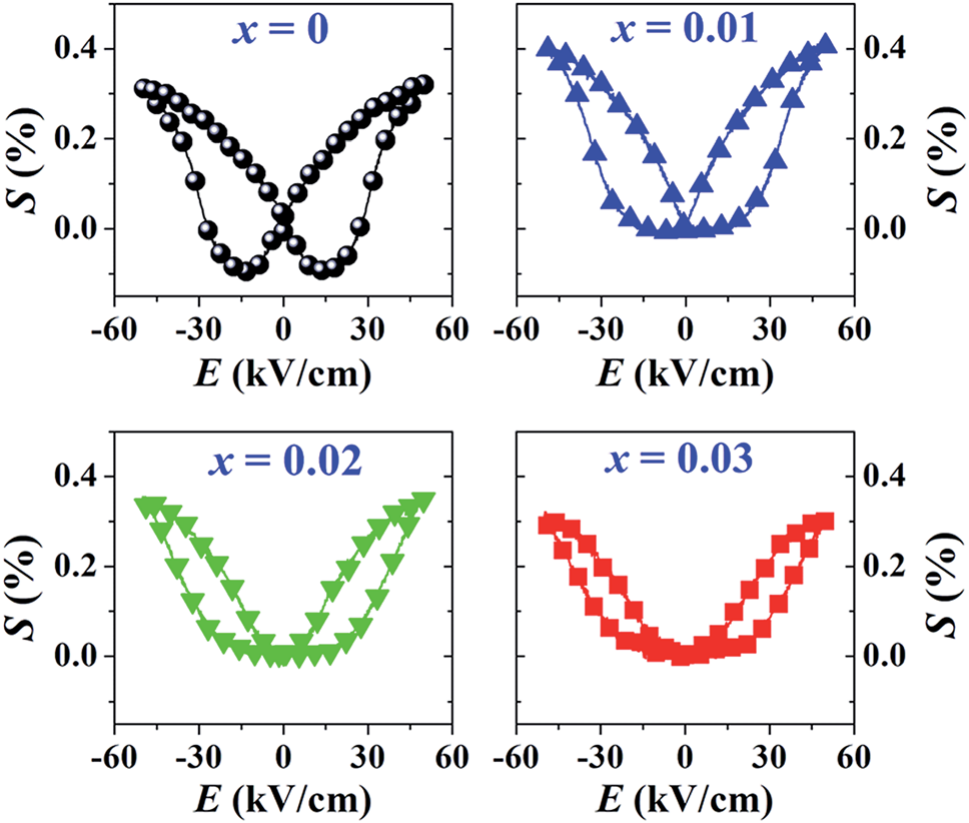
Bipolar strain–electric field (*S*–*E*) loops of the (1 − *x*)[BNKT–0.03BSrT]–*x*BFT ceramics where *x* = 0–0.03, measured at RT under an electric field of 50 kV cm^−1^ and a frequency of 0.1 Hz.

The *P*–*E* hysteresis and bipolar strain–electric field (*S*–*E*) loops measured at room temperature (RT) and electric field of 50 kV cm^−1^ are shown in Fig. 5 and 6, respectively. The related values are also listed in Tables 2 and 3. The *x* = 0 ceramic displays a small pinched loop with the maximum values of *P*_max_ = 37.70 μC cm^−2^, *P*_r_ = 21.74 μC cm^−2^ and *E*_c_ = 12.87 kV cm^−1^. The *S*–*E* curve for this sample also exhibits a butterfly-shaped loop. The *S*_max_ and 
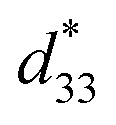
 of this ceramic are 0.33% and 650 pm V^−1^, respectively. The ferroelectric loop indicates a ferroelectric domain switching at *E*_c_, where the largest *S*_neg_ is −0.11%. However, this ceramic sample should contain a mixed phase between FE and ER, since the *P*–*E* loop presents a small pinched shape. For the *x* = 0.01 ceramic, however, the *P*–*E* hysteresis loop exhibits a more pinched loop with a decrease of *P*_r_ from 21.74 μC cm^−2^ to 8.15 μC cm^−2^ and *E*_c_ from 12.87 kV cm^−1^ to 7.34 kV cm^−1^. The hysteresis loop shows a clear pinching-type character with further increasing BFT content, similarly observed in previous works.^6,35^ The pinched *P*–*E* loops corresponded with the two current peaks (denoted as “1” and “2”) in the *I*–*E* curves (Fig. 5), where it has been suggested that this evidence indicates a formation of ER phase.^14,36^

**Table 2:** Ferroelectric and energy storage density properties of the (1 − *x*)[BNKT–0.03BSrT]–*x*BFT ceramics

*x*	*P*_max_ (μC cm^−2^)	*P*_r_ (μC cm^−2^)	*E*_c_ (kV cm^−1^)	*W* a (J cm^−3^)	*W* b (J cm^−3^)	*W* c (J cm^−3^)	*η* a (%)	*η* b (%)	*η* c (%)
0	37.70	21.74	12.87	0.20	0.54	0.60	20.0	69.5	81.2
0.01	35.65	8.15	7.34	0.37	0.61	0.61	38.6	80.0	87.4
0.02	32.78	4.26	5.66	0.45	0.63	0.62	51.7	87.9	92.0
0.03	29.18	3.15	4.77	0.49	0.65	0.63	60.7	90.4	92.1

a Ferroelectric data obtained at high temperature of 25 °C and a frequency of 1 Hz.

b Ferroelectric data obtained at high temperature of 120 °C and a frequency of 1 Hz.

c Ferroelectric data obtained at high temperature of 150 °C and a frequency of 1 Hz.

**Table 3:** Piezoelectric properties of the (1 − *x*)[BNKT–0.03BSrT]–*x*BFT ceramics

*x*	*S*_max_ (%)	*S*_neg_ (%)	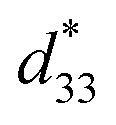 (pm V^−1^)	*H* (%)	*Q*_33_ (m^4^ C^−2^)	*d*_33_ (pC N^−1^)
0	0.33	−0.11	650	78.9	0.0376	172
0.01	0.42	0	840	55.0	0.0404	38
0.02	0.37	0	722	44.6	0.0383	29
0.03	0.32	0	654	39.3	0.0368	28

**Fig. 7:**
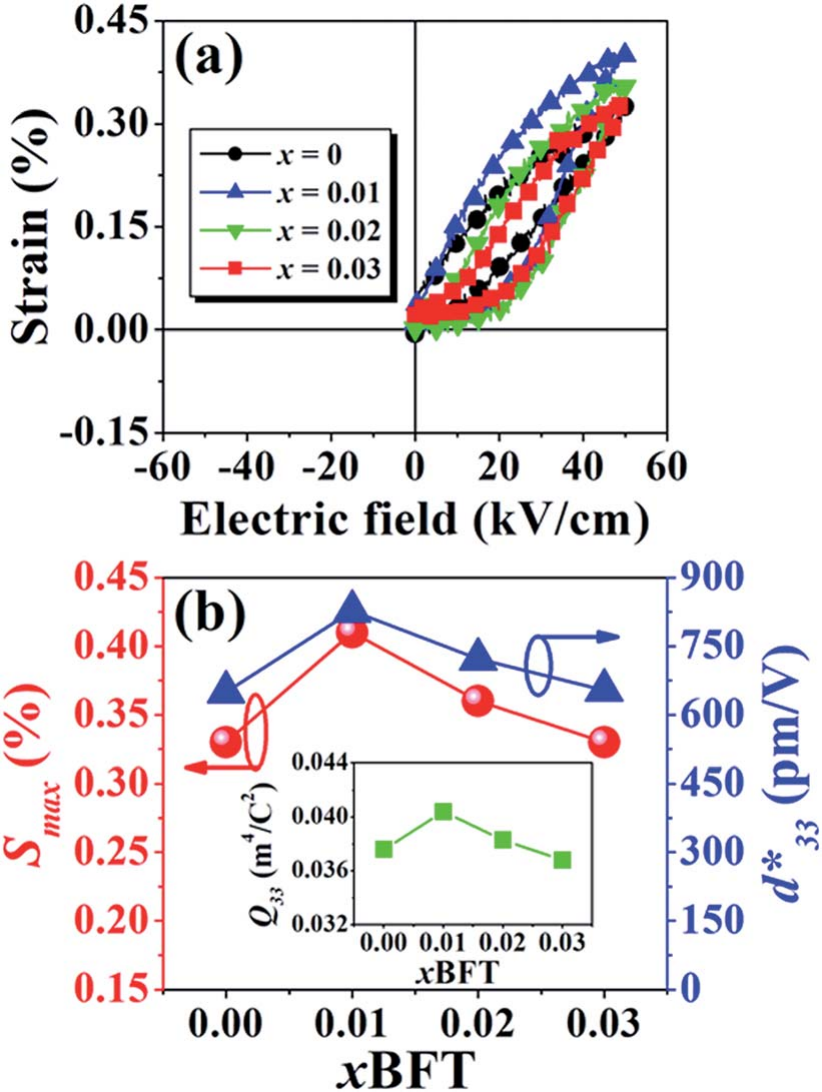
Plots of (a) unipolar strain–electric field (*S*–*E*) loops of the (1 − *x*)[BNKT–0.03BSrT]–*x*BFT ceramics where *x* = 0–0.03, measured at RT under an electric field of 50 kV cm^−1^ and a frequency of 0.1 Hz and (b) plots of the maximum strain (*S*_max_) and normalized strain coefficient 
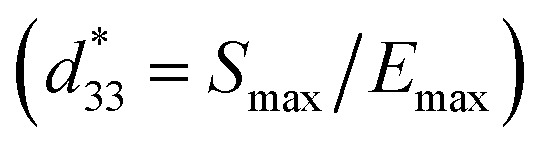
 as a function of BFT content (inset: *Q*_33_ value as a function of BFT content).

The more pinched *P*–*E* loops result also corresponds to a large electric field-induced strain response with *S*_max_ of 0.42% and 
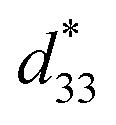
 of 840 pm V^−1^ along with a drastic reduction in *S*_neg_ from −0.11% to 0% for the *x* = 0.01 ceramic in bipolar *S*–*E* loops. This enhancement is also confirmed by the unipolar *S*–*E* loops, as shown in Fig. 7(a). It is seen that the unipolar *S*–*E* loops for all compositions show a similar trend as compared to that of the bipolar *S*–*E* loops, where the maximum strain value of the unipolar strain is 0.42% for the *x* = 0.01 ceramic (Fig. 7(b)). The pinching of the *P*–*E* hysteresis loops with rapid reductions of *P*_r_ and *E*_c_ can be correlated with the increase of *S*_max_ along with a drastic reduction in *S*_neg_. This may be related with a phase transition from a ferroelectric (FE) to an ergodic relaxor (ER) phase, accompanied by the disruption of long-range ferroelectric order with increasing BFT content.^6^ Normally, the free energy of FE and ER phases is comparable under zero field, thus it can be easily induced by an external electric field to become saturated.^36^ Therefore, ER phase can be transformed reversibly into FE phase by an external electric field. This behaviour consists with the work done by Dong *et al.*^40^ who also reported that the (1 − *x*)(0.8Bi_1/2_Na_1/2_TiO_3_–0.2Bi_1/2_K_1/2_TiO_3_)–*x*BiMg_2/3_Nb_1/3_O_3_ or (BNT–BKT)–BMN system produced a large strain response when a small amount of BMN was added into the BNT–BKT ceramic. The addition of 2 mol% BMN into the BNT–BKT ceramic shows a pronounced ergodic relaxor (ER) characteristic, which is confirmed by the absence of *S*_neg_, the pinched *P*–*E* loop and the double current peaks of *I*–*E* loop. However, in the present work when *x* > 0.01, the strain is reduced gradually. Therefore, the small amount of BFT additive has effects on the piezoelectric properties of the BNKT–0.03BSrT ceramics. It should be noted that the *S*_max_ in this work (*x* = 0.01) is considered high when compared with other lead-free piezoelectric ceramics (see Table 4).^18,41–48^ Furthermore, the bipolar strain loops for the present work exhibit a low anti-symmetry in their shape compared to many previous works for the giant strain response which showed high anti-symmetry.^42,45^ In the present work, an average maximum strain (*S̄*_max_) was calculated to be used as a parameter to check for overall averaged maximum strain of the bipolar strain loop, where *S̄*_max_ averaged from maximum strain between the right and left hand sides of the bipolar strain loop. The result is also shown in Table 4. The obtained *S̄*_max_ for the present work is considered high for the lead-free piezoelectric ceramics.

**Table 4:** Comparison of *S*_max_ and 
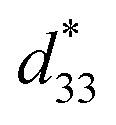
 values of the (1 − *x*)[BNKT–0.03BSrT]–*x*BFT (*x* = 0.01) with other lead-free ceramics

Systems	*S*_max_ (%)	*S̄*_max_ (%)	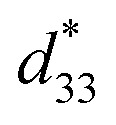 (pm V^−1^)	*E*_max_ (kV cm^−1^)	Ref.
0.99[BNKT–0.03BSrT]–0.01BFT	0.42	0.42	827	50	This work
BNKT–BST–La_*x*_	0.39	0.39	650	60	18
Bi_0.5_(Na_0.78_K_0.22_)_0.5_(Ti_1−*x*_Z_*x*_)O_3_, *x* = 0.03	0.43	0.43	614	70	41
Bi_1/2_(Na_0.8_K_0.2_)_1/2_](Ti_1−*x*_Ta_*x*_)O_3_, *x* = 0.015	0.62	0.42	1240	50	42
BNKT–BST–Nb_*x*_, *x* = 0.02	0.38	0.38	634	60	43
Bi_0.5_(Na_0.78_K_0.22_)_0.5_TiO_3_–0.01(Bi_0.5_La_0.5_)AlO_3_	0.40	0.40	579	70	44
((Bi_1/2_(Na_0.84_K_0.16_)_1/2_)_0.96_Sr_0.04_)(Ti_1−*x*_Nb_*x*_)O_3_, *x* = 0.025	0.70	0.39	1400	50	45
Bi_0.5_(Na_0.78_K_0.22_)_0.5_TiO_3_–0.03BiFeO_3_	0.37	0.36	676	50	46
Bi_0.5_(Na_0.80_K_0.20_)_0.5_TiO_3_–0.05Ba(Ti_0.90_Sn_0.10_)O_3_	0.36	0.36	649	55	47
[0.93(Bi_0.5_Na_0.5_)TiO_3_–0.07BaTiO_3_]–Pr	0.43	0.42	770	50	48

The *Q*_33_ was determined from the slope of *S vs. P*^2^ curve.^49,50^ The plotting of *Q*_33_ value as a function of BFT content is shown in inset of Fig. 7(b). The highest *Q*_33_ of 0.040 m^4^ C^−2^ is noted for the *x* = 0.01 ceramic. It is evident that the currently presented *Q*_33_ value is highly competitive to many materials.^51–58^ In addition, a comparison of the room temperature *Q*_33_ and normalized *Q*_33_ (*Q*_33_/*E*) values with other lead-based and lead-free BNT-based electrostrictive materials are summarized in Table 5.^51–58^ It should be noted that the *Q*_33_/*E* value for the present work is very high for both lead-based and lead-free BNT-based piezoelectric materials. Therefore, the (1 − *x*)[BNKT–0.03BSrT]–*x*BFT compound may be a promising candidate for new electromechanical devices due to its giant *Q*_33_ value.

**Table 5:** Comparison of *Q*_33_ and *Q*_33_/*E* values of the (1 − *x*)[BNKT–0.03BSrT]–*x*BFT (*x* = 0.01) with other lead and lead-free ceramics

Systems	*S*_max_ (%)	*Q*_33_ (m^4^ C^−2^)	*Q*_33_/*E*_max_ (m^5^ C^−2^ V) × 10^−9^	*E* (kV cm^−1^)	Ref.
0.99[BNKT–0.03BSrT]–0.01BFT	0.42	0.0404	8.08	50	This work
Pb(Mg_1/2_Nb_1/2_)O_3_	—	0.023	—	—	51
PLZT 8/65/35	0.16	0.015	5.00	30	52
0.96BNKT–0.04BNiT	0.38	0.0250	4.17	60	53
0.95BNKT–0.05BNiT	0.32	0.0300	5.00	60	53
N44B48T94–6BT [or (Na_*y*_,Bi_*z*_)Ti_1−*x*_O_3_(1 − *x*)–*x*BaTiO_3_]	0.48	0.0260	3.71	70	54
8% Sn-doped Bi_1/2_(Na_0.82_K_0.18_)_1/2_TiO_3_	0.14	0.0230	3.83	60	55
BNKT100*y*–*x*KNN (*y* = 0.20, *x* = 0.16)	0.10	0.0250	3.12	80	56
(0.94 − *x*)BNT–0.06BT–*x*KNN, *x* = 0.20	0.09	0.0260	3.25	80	57
Bi_0.5−*x*_La_*x*_Na_0.40_K_0.10_Ti_0.98_Zr_0.02_O_3_, *x* = 0.02	0.42	0.0360	6.00	60	58

Normally for the actuator applications, a large strain with low hysteresis is necessary. The degree of strain hysteresis (*H*) can be determined from the following equation:^38,59^2
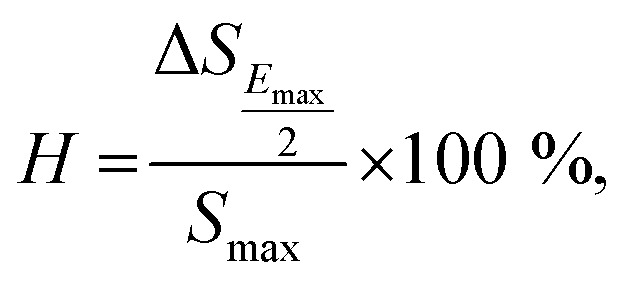
where the Δ*S*_*E*_max_/2_ is the strain deviation during the application and removal of the field which is carried out at half of the maximum electric field. The *H* values of all ceramics are summarized in Table 3. It was found that the *H* decreases with increasing BFT concentration. The lowest hysteresis of 39.3% is obtained at *x* = 0.03 @ 50 kV cm^−1^, which reveals that BFT doping obviously reduces the strain hysteresis. A comparison of the *H* value for selected ceramic samples with other lead-free ceramics is shown in Table 6.^32,60–66^ The result clearly demonstrates that the *H* value of the present work is low when compared to that of many previous works (for the lead-free piezoceramics with 
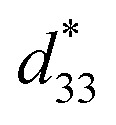
 value > 450 pm V^−1^). This suggests that a BFT modifier can improve the actuating performance.^38^

**Table 6:** Comparisons of the degree of strain hysteresis (*H*) value of the (1 − *x*)[BNKT–0.03BSrT]–*x*BFT (*x* = 0.01 and *x* = 0.03) with other lead-free ceramics

Systems	*H* (%)	*S*_max_ (%)	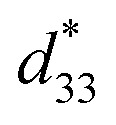 (pm V^−1^)	Ref.
0.99[BNKT–0.03BSrT]–0.01BFT	55.0	0.42	840	This work
0.97[BNKT–0.03BSrT]–0.03BFT	39.3	0.32	654	This work
Bi_1/2_(Na_0.82_K_0.12_)_1/2_Ti_1−*x*%_(Fe_0.5_Nb_0.5_)_*x*%_O_3_, *x* = 5%	57.0	0.46	660	32
BNKT20–1.5SZ	51.6	0.39	488	60
BNT–BKT–5La	60.3	0.38	857	61
BNT–25ST	45.8	0.29	650	62
BNKT–3 mol% CZ	49.0	0.37	603	63
BNKT–2 mol% BCZ	25.0	0.30	549	63
(Ho,Sb)-modified (Bi_0.5_Na_0.5_)_0.945_Ba_0.065_TiO_3_	39.0	0.37	463	64
BNBT6.5–100*x*ES, *x* = 0.50%	39.0	0.40	500	65
BNT–BKT–*x*BZT, *x* = 0.02	40.0	0.32	503	66

### Polarization-temperature analysis

**Fig. 8:**
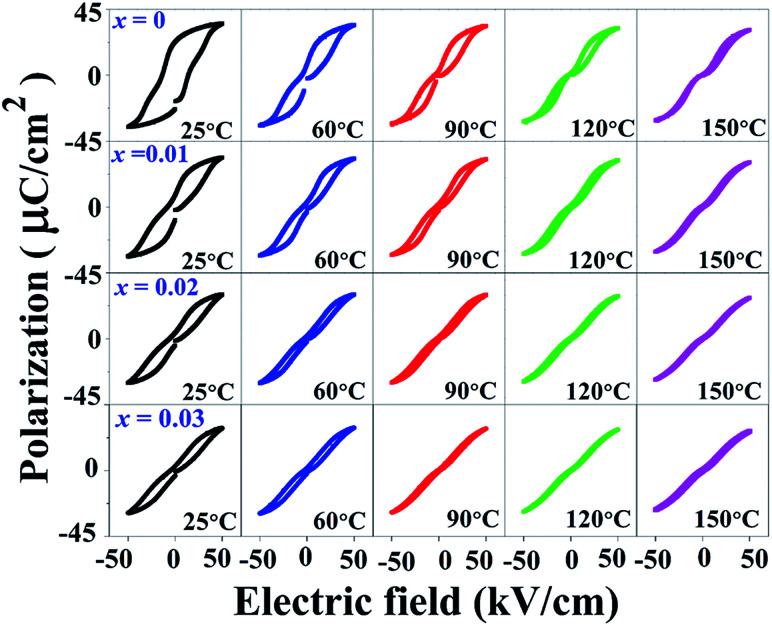
Temperature dependence on polarization–electric field (*P*–*E*) hysteresis loops of the (1 − *x*)[BNKT–0.03BSrT]–*x*BFT ceramics where *x* = 0–0.03, measured under an electric field of 50 kV cm^−1^ and a frequency of 1 Hz.

Temperature dependence of polarization–electric field (*P*–*E*) hysteresis loops of the (1 − *x*)[BNKT–0.03BSrT]–*x*BFT ceramics where *x* = 0–0.03, measured under an electric field of 50 kV cm^−1^ and a frequency of 1 Hz is shown in Fig. 8. It can be seen that the *P*–*E* hysteresis loop for the *x* = 0 ceramic shows slightly pinched loops at RT (25 °C). When the temperature increases to 120–150 °C, the *P*–*E* hysteresis loops become more pinched, confirming that the ferroelectric to relaxor phase transition is induced by thermal activation, thus leaving an ergodic relaxor (ER) state at zero electric field.^67–69^ On the other hand, the modified ceramics (*x* ≥ 0.01), exhibit an ER characteristic at RT. With further increasing temperature, the drastic decrease in both *P*_r_ and *E*_c_ values may also be related to the onset of strong ergodicity.^68^ Thus, both chemical modification and temperature can disrupt the FE long-range order leading to a decrease in the polarization states.^68^ This phenomenon agrees with that observed in many lead-free piezoelectric materials.^40,69^ Dong *et al.*^40^ also studied the temperature dependence of *P*–*E* hysteresis loops of the lead-free (1 − *x*)(0.8Bi_1/2_Na_1/2_TiO_3_–0.2Bi_1/2_K_1/2_TiO_3_)–*x*BiMg_2/3_Nb_1/3_O_3_ ceramics. With increasing temperature, the *P*_r_ and *E*_c_ values decrease gradually while *P*_max_ slight changed and the *P*–*E* hysteresis loop showed severe pinches. This indicated the presence of an ergodic relaxor (ER) state, *i.e.* there exists a temperature-caused ferroelectric-to-relaxor phase transformation in their work.

### Energy storage density

The polarization and dielectric breakdown strength are two important parameters related to the energy storage performance. As is known to all, the BNT–BKT ceramics with high *P*_r_ of 34 μC cm^−2^ and low breakdown field (*E*_b_ ∼ 50 kV cm^−1^) are difficult to a satisfactory characteristic for high energy storage applications.^70^ Therefore, the design of BNT–BKT energy storage materials with low *P*_r_, large *P*_max_ as well as the improvement of *E*_b_ become two critical issues.

**Fig. 9:**
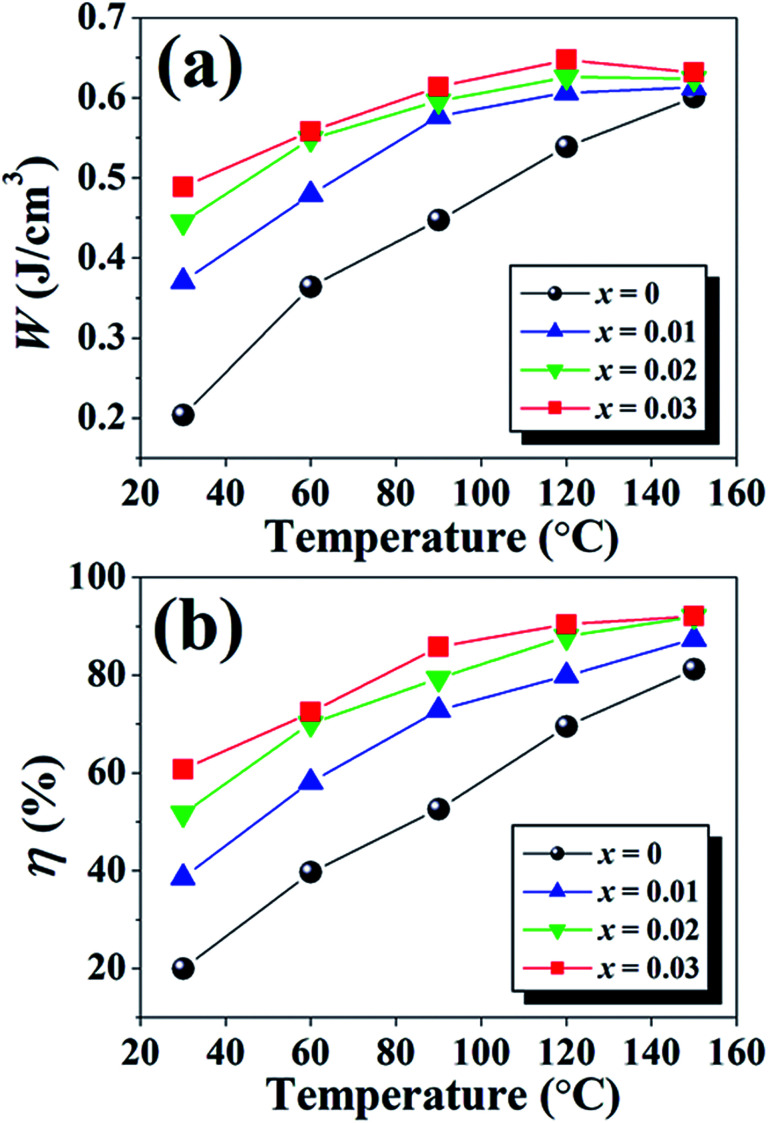
Plots of (a) energy storage density (*W*) and (b) energy storage coefficient (*η*) as a function of temperature of the (1 − *x*)[BNKT–0.03BSrT]–*x*BFT ceramics.

To evaluate the capability of the studied ceramics for energy storage applications, the energy storage density (*W*) was calculated from the *P*–*E* hysteresis loop as shown in the following equation:^70,71^3
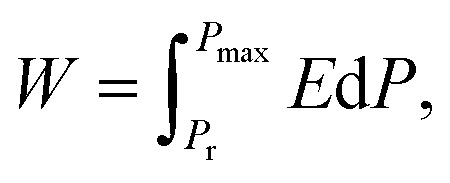
where *E* is the electric field, *P* the polarization, *P*_max_ the maximum polarization, and *P*_r_ is the remnant polarization. For practical applications, higher energy storage efficiencies (*η*) are required. The *η* value is calculated by using the following formula:^68,72^4
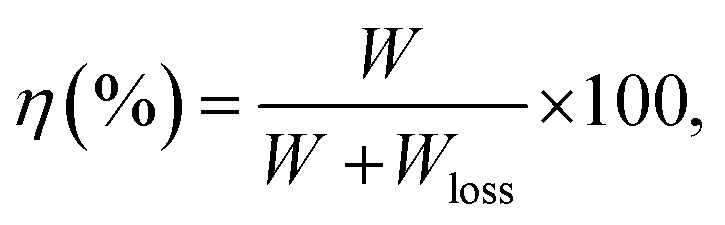
where energy loss density is denoted as *W*_loss_. In this work, the energy storage density properties are summarized in Table 2. At RT, the *W* and *η* of the *x* = 0 ceramic are 0.20 J cm^−3^ and 20.0%, respectively. In this composition, the high *E*_c_ and low *P*_max_ − *P*_r_ values are observed, which led to a low energy storage density, high energy loss, and low efficiency.^72^ The *x* = 0.01 ceramic with a pinched *P*–*E* hysteresis loop shows relatively high *W* = 0.37 J cm^−3^ and *η* = 38.6% values when compared with those observed for the *x* = 0 ceramic. This improvement can be related with the decreases in *P*_r_, and slight invariant *P*_max_ with the degradation of the long range FE order and a relatively easy reorientation of the micro-domains.^72^ The *W* and *η* values increase with increasing BFT content and exhibit the maximum values of 0.49 J cm^−3^ and 60.7% at the electric field of 5 kV mm^−1^ for the *x* = 0.03 ceramic. These values are also higher than the value obtained for the *x* = 0 sample by ∼145% and 203%, respectively. In addition, the *W* and *η* values as a function of temperature of the (1 − *x*)[BNKT–0.03BSrT]–*x*BFT ceramics are presented in Fig. 9. It can also be seen that the *W* and *η* increase with increasing temperature for all compositions and reach the maximum value of ∼0.65 J cm^−3^ and 90.4% at 120 °C for the *x* = 0.03 ceramic. These obtained *W* values are close to the value obtained in Bi_0.48_La_0.02_NKTZ ceramics (*W* ∼ 0.63 J cm^−3^, at RT) as reported by Butnoi *et al.*^58^

A comparison of normalized energy storage density (*W*/*E*) with the previous reports is presented in Table 7.^38,70,72–81^ The comparison of *W*/*E* value with previous results clearly shows the superiority of this composition (*x* = 0.03) in terms of high *W*/*E* = 0.13 μC mm^−2^ and *η* = 90.4%. The obtained *W*/*E* value is high for Bi-based piezoelectric ceramics. Based on the obtained results, it is suggested that these synthesized ceramics are promising materials for actuator, high electric power and energy storage applications.^72,77^

**Table 7:** Comparison of *W* and *W*/*E* of the (1 − *x*)[BNKT–0.03BSrT]–*x*BFT ceramics (*x* = 0.03) with other lead-free ceramics

Systems	*W* (J cm^−3^)	*E* (kV mm^−1^)	*W*/*E* (μC mm^−2^)	*η* (%)	Ref.
0.97[BNKT–0.03BSrT]–0.03BFT @ 120 °C	0.65	5	0.13	90.4	This work
0.96BNKT–0.04MN	0.65	7	0.09	34.2	38
0.92BNKT–0.08AN	1.41	10.5	0.13	—	70
Nb-modified 0.96(Bi_0.5_Na_0.84_K_0.16_TiO_3_)–0.04SrTiO_3_	1.00	6	0.17	>70	72
0.94Bi_0.47_Na_0.47_Ba_0.06_TiO_3_–0.06KNbO_3_	0.89	10	0.09	—	73
(0.9 − *x*)[0.92Bi_0.5_Na_0.5_TiO_3_–0.08BaTiO_3_]–*x*SrZrO_3_–0.10NaNbO_3_, *x* = 0.08	0.95	11	0.09	66.0	74
0.92(0.65BaTiO_3_–0.35Bi_0.5_Na_0.5_TiO_3_)–0.08Na_0.73_Bi_0.09_NbO_3_	1.70	17.2	0.10	82.0	75
BNTBT–0.20NBN	1.36	13.6	0.10	73.9	76
(Bi_0.5_Na_0.5_)TiO_3_–0.10KNbO_3_	1.17	10.4	0.11	—	77
0.7SrTiO_3_–0.3(0.65BaTiO_3_–0.35Bi_0.5_Na_0.5_TiO_3_)	1.40	19.6	0.07	90.0	78
0.95(BNTBT)–0.05KN	0.64	6	0.11	84	79
0.99[0.7BNT–0.3BST]–0.01NN	1.03	8.5	0.12	85.8	80
(BNT–6BT)–NBN	1.40	14.2	0.11	66.3	81

## Conclusions

In this work, the BNKT–0.03BSrT doped with BFT ceramics have been successfully synthesized by a solid-state mixed oxide method. The coexistence of rhombohedral and tetragonal phases is present throughout the entire compositional range, while a dominant tetragonal-rich phase is observed at a higher BFT content. The *x* = 0.01 ceramic shows the high *S*_max_ of 0.42%, 
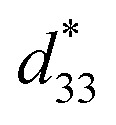
 of 840 pm V^−1^, and high *Q*_33_ of 0.0404 m^4^ C^−2^ (at RT) with a very high *Q*_33_/*E* (8.08 × 10^−9^ m^5^ C^2^ V^−1^). The maximum energy storage density (*W* = 0.65 J cm^−3^ @ 120 °C) and energy storage efficiency (*η* = 90.4% @ 120 °C) with large improvements are obtained for the *x* = 0.03 ceramic. The studied results indicate that BFT enhances the electric field-induced strain, electrostrictive coefficient and energy storage density performances in BNKT-based ceramics. These ceramics can be considered promising candidates for actuator and high electric power pulse energy storage applications.

## Conflicts of interest

There are no conflicts to declare.

## Supplementary Material
